# A Smith Predictor Modified with a Pseudo Feedforward Control for the Charge-Coupled Device-Based Optoelectronic Tracking System

**DOI:** 10.3390/s24175546

**Published:** 2024-08-27

**Authors:** Keran Deng, Juan Tan, Piao Chen, Shige Zhang, Ke Wang, Yong Luo

**Affiliations:** 1 Chongqing Key Laboratory of Photo-Electric Functional Materials and Laser Technology, College of Physics and Electronic Engineering, Chongqing Normal University, Chongqing 401331, China; 20200089@cqnu.edu.cn (K.D.); imaons@126.com (K.W.); 2 CMA Institute for Development and Programme Design, Beijing 100081, China; tanjuan@cma.gov.cn (J.T.); chenpiao233@163.com (P.C.); zhangshige805524@cma.cn (S.Z.); 3 School of Automation, Nanjing University of Information Science and Technology, Nanjing 210044, China

**Keywords:** smith predictor, pseudo feedforward control, fast steering mirrors, high-precision optoelectronic tracking system

## Abstract

In the high-precision optoelectronic tracking system (OTS) based on a charge-coupled device (CCD), the boresight error extracted from the tracking image contains an undeniable delay, which directly limits the control bandwidth of visual tracking. High bandwidth means high response speed and tracking accuracy. Generally, a model-based delay compensation control method called the Smith predictor is utilized to separate time delay from the closed loop to promote the control bandwidth. However, due to the existence of errors between the established model and the real object, the improvement in the bandwidth is still limited to ensure system stability, resulting in insufficient tracking performance. In this paper, to solve the problem, a Smith predictor modified with pseudo feedforward control for the OTS is proposed. The experimental results demonstrate that the proposed method achieves significant improvements in tracking performance, reducing the maximum residual error at 1 Hz from 365 arcseconds (using the classic Smith predictor) to 283 arcseconds, a 22.5% improvement. Across the main frequency band (0.2 Hz to 2 Hz), the residual errors were consistently lower using the proposed method.

## 1. Introduction

The optoelectronic tracking system (OTS) based on a charge-coupled device (CCD) is widely used in precision control fields such as astronomical observation, laser communication, monitoring and search [1,2,3,4]. The control unit of the OTS drives the lens to always follow the moving target through the boresight error value. The higher the closed-loop bandwidth, the stronger the target tracking ability. However, the imaging of the target and the extraction of the boresight error consume significant time, which can cause significant delays in closed-loop control [5]. Delays can rapidly reduce the system’s phase margin, directly limiting the amplitude of the controller and resulting in a significant decrease in the control bandwidth [6]. In order to reduce the limitations in controller bandwidth on control performance, previous researchers mainly adopted methods such as improving controller types and directly compensating for time delays.

Traditional PID controllers can calibrate the system to a type one system [7]. Their ability to suppress low-frequency errors is insufficient under limited bandwidth. To solve the problem, a PID-I controller was proposed to significantly improve the low-frequency error suppression ability by adding an additional integration link. However, this enhancement comes at the cost of a significant decrease in system stability [8]. In order to better balance the relationship of the system’s type and the control stability, the fractional order control method was introduced into optoelectronic tracking systems, which sacrifices less margin to achieve sufficient improvement in error suppression ability [9]. In addition to improving the design of feedback controllers, an additional feedforward control is also a commonly used way to promote the performance due to its advantage of upgrading the type of control system without affecting system stability [10]. In order to obtain the current target position signal for feedforward, it is necessary to combine the boresight error and the telescope motion state for prediction [11]. It should be pointed out that due to the low bandwidth of trajectory prediction, feedforward often can only reduce the impact of delay on tracking performance in the low frequency range.

Unlike the methods of improving the system type, a time delay compensation control method called the Smith predictor can compensate for the impact of time delay on stability through the assistance of a reference model, which is widely used in industrial control systems with large time delays [12,13,14]. The basic principle of the Smith predictor is to provide early feedback through a model output without time delay, approximately removing the delay from the closed loop to improve the control bandwidth [15]. Although the Smith predictor was proposed earlier and has the disadvantage of being easily affected by parameter changes, it is still an effective control scheme widely used in systems with delay. The application of the Smith predictor in optoelectronic tracking systems began in recent years and has achieved beneficial results in improving the tracking and anti-interference performance of the system. Cao first applied the Smith predictor to a fast mirror tracking system with high model linearity, which significantly improved the control bandwidth and accuracy of the system [16]. In order to improve the anti-interference performance of delay systems, Ren proposed an improved Smith predictor with disturbance observation feedforward, which can simultaneously compensate for time delay and suppress external interference [17]. In order to further eliminate the impact of time delay separating from the closed loop on performance, a trajectory prediction structure based on Kalman filtering is added to the Smith predictor, which can almost completely compensate for the time delay in the low frequency [18]. It should be pointed out that although the above Smith predictor methods can improve control bandwidth by separating time delays, the control bandwidth of the system is still limited to a certain range due to the problem of inaccurate reference models, resulting in insufficient tracking performance by the system.

In this paper, to address the influence of the mismatch between the established model and the real model on system performance improvement, a novel Smith predictor modified by pseudo feedforward control for the OTS is proposed. In order to reduce the impact of parameter perturbations on the performance of the Smith predictor, the Smith predictor is first constructed based on a velocity closed-loop model with stronger robustness. Then, considering that feedforward is a good way to continue improving performance when feedback control capability is limited and the model of the object is available, an extra pseudo feedforward branch is added inside the structure of the Smith predictor to improve the error suppression ratio. Finally, the paper analyzed the impact of delay mismatch on control stability and provided the optimal design conditions for feedforward and feedback controllers. In order to verify the effectiveness of the proposed method in this paper, an optoelectronic tracking system based on fast steering mirrors was established. The experimental results show that the proposed method can significantly improve the low-frequency tracking ability of the system under bandwidth constraints.

This paper is organized as below. Section 2 presents a detailed introduction to the CCD-based opto-electric tracking system, mainly describing the control structure and the classic Smith predictor. Section 3 gives out the structure of the modified Smith predictor by pseudo feedforward and discusses the promotion of tracking performance. Section 4 gives the constraints on the parameters and provides the optimal parameter selection when delay mismatch is faced. Section 5 sets up experiments to confirm that the proposed method is effective. Concluding remarks are presented in Section 6.

## 2. The Classic Smith Predictor for the Optoelectronic Tracking System

**Figure 1:**
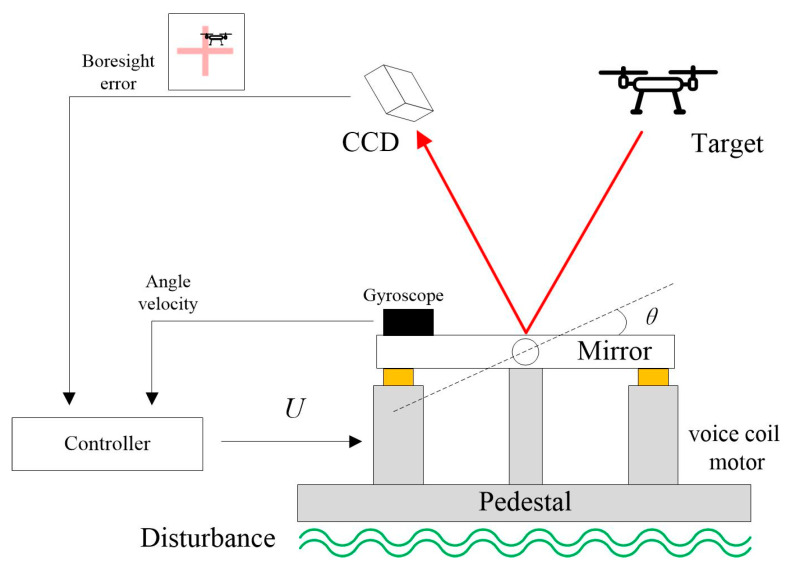
The structure of CCD-based OTS. *U* represents the control input for the platform and *θ* denotes the deflection angle of the mirror.

The configuration of the CCD-based OTS is illustrated in Figure 1. The target light is reflected through a fast steering mirror and enters the CCD for imaging. The controller drives the deflection of the fast steering mirror based on the boresight error calculated by the image processor to track the target and suppress the external disturbance. Usually, the mirror is driven by voice coil motors with high bandwidth, linearity, and precision. In order to ensure the inertial stability of the system, additional inertial navigation devices are usually added to the OTS to improve control performance.

**Figure 2:**
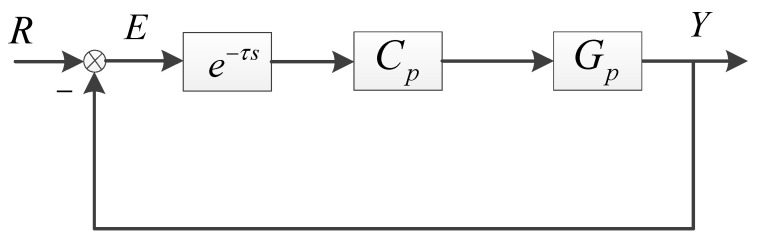
The position-loop feedback control of the OTS.

The control structure of the boresight error-based position-loop feedback is presented in Figure 2. $G_{p}$ is the transfer function of the fast steering mirror. $C_{p}$ refers to the position controller. $e^{-\tau s}$ represents the transfer function of delay caused by image processing, where τ is the delay time. E refers to the boresight error. R is the input of the system, representing the actual deviation angle of the target relative to the boresight. Y is the output of the photoelectric tracking system, representing the actual deflection angle of the fast mirror following the target. Since the negative effect of delay on tracking and anti-disturbance is consistent, we only investigate the tracking error without external disturbance. The tracking error transfer function of the position-loop feedback is exhibited as Equation (1).

(1) $$S_{0}=\frac{E}{R}=\frac{1}{1+C_{p}G_{p}e^{-\tau s}}$$

**Figure 3:**
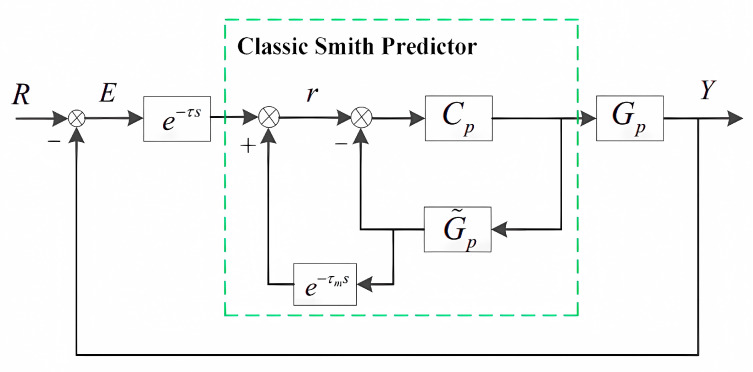
The classic Smith predictor control for OTS.

From the tracking error transfer function $S_{0}$, it is concluded that the larger the amplitude of the controller $C_{p}$, the higher the bandwidth and the smaller the tracking error. However, due to the direct phase lag caused by delay, the gain of the controller is limited to a small range, seriously limiting the accuracy of the system. To reduce the effect of time delay, the classic delay-compensated controller (also called the Smith predictor) is added to the position-loop feedback control, as shown in Figure 3. The dotted frame is the Smith predictor, in which $e^{-\tau_{m}s}$ is the estimation of the CCD delay and ${\tilde{G}}_{p}$ is an object model obtained through frequency identification. The tracking error transfer function in Figure 3 is exhibited as Equation (2).

(2) $$S_{1}=\frac{E}{R}=\frac{1+C_{p}{\tilde{G}}_{p}\left(1-e^{-\tau_{m}s}\right)}{1+C_{p}{\tilde{G}}_{p}+C_{p}\left(G_{p}e^{-\tau s}-{\tilde{G}}_{p}e^{-\tau_{m}s}\right)}$$

If the model identification of the platform and delay is very accurate, then $G_{p}\approx {\tilde{G}}_{p}$ and $\tau \approx \tau_{m}$ can be obtained. Additionally, since $\tau_{m}\ll 1$ is common at low frequencies, we can obtain $e^{-\tau_{m}s}\approx 1$ at this frequecy domain. Therefore, Equation (2) can be simplified into the following form.

(3) $$S_{1}\approx \frac{1}{1+C_{p}{\tilde{G}}_{p}}$$

Comparing $S_{0}$ and $S_{1}$, it can be concluded that the closed-loop transfer function will no longer contain time-delay components and the delay is approximately shifted outside the closed-loop system. At this point, the gain of the controller can be taken as a larger value, which means that the system will have higher bandwidth and smaller tracking error.

It should be pointed out that the improvement in the low-frequency tracking performance of the delay system by the Smith predictor is foreseeable, but the controller gain of the system still cannot be achieved significantly because such matching between the real object and the constructed model in practice can seldom be achieved. After considering the mismatch, the gain of the controller is limited to guarantee the system stability and the improvement effect of the Smith predictor on the system’s tracking performance will be determined by the degree of model mismatch between the real object and the model. Due to the imprecise modeling in the mid-to-high frequency region and the presence of parameter perturbations during system operation, it is also difficult to obtain sufficient tracking accuracy using the classic Smith predictor-based methods.

## 3. The Modified Smith Predictor by Pseudo Feedforward Control

**Figure 4:**
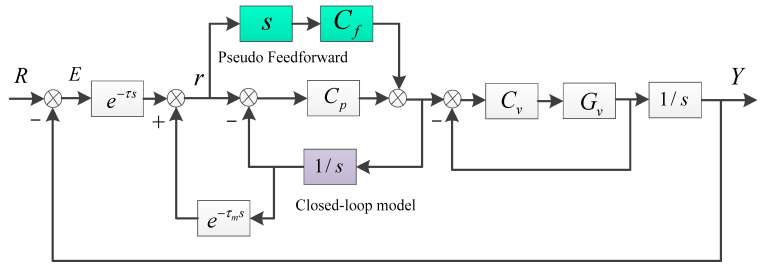
The modified Smith predictor by pseudo feedforward control.

In order to further improve the tracking performance of the system, the modified Smith predictor by pseudo feedforward control is proposed, which is exhibited as Figure 4. Firstly, the improved Smith predictor is based on a closed-loop model which will be less affected by parameter perturbations compared to an open-loop model. The added pseudo feedforward link is used to improve the transmission characteristics of the system for better tracking performance when feedback performance is limited and the model is available.

**Figure 5:**
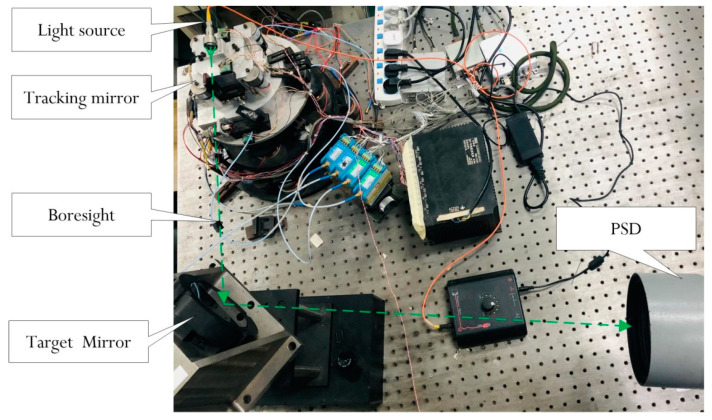
Experimental apparatus.

In Figure 5, a velocity loop is first constructed inside the position loop. Due to the high sampling rate of the velocity loop, a closed-loop model close to an integral link can be obtained, which is very conducive to the construction of the Smith predictor. In the pseudo feedforward branch, $C_{f}$ is the feedforward controller to be designed to ensure the system’s stability. The tracking error transfer function in Figure 5 is exhibited as Equation (4), in which $V=C_{v}G_{v}/\left(1+C_{v}G_{v}\right)$ is the velocity closed-loop transfer function.

(4) $$S_{2}=\frac{E}{R}=\frac{\left(1-C_{f}e^{-\tau_{m}s}\right)+C_{p}\frac{1}{S}\left(1-e^{-\tau_{m}s}\right)}{1+C_{p}\frac{1}{S}+C_{p}\frac{1}{S}\left(Ve^{-\tau s}-e^{-\tau_{m}s}\right)+C_{f}\left(Ve^{-\tau s}-e^{-\tau_{m}s}\right)}$$

We focus on the comparison of the tracking error transfer functions of the classic Smith predictor and the modified one. When s→0, $e^{-\tau_{m}s}=e^{-\tau s}\to 1$ and V→1. Therefore, if $C_{f}$ is set to 1, the following result can be concluded.

(5) $$s\to 0,\;\left|S_{1}\right|\to \frac{1}{\left|1+C_{p}\frac{1}{s}\right|}$$

(6) $$s\to 0,\;\left|S_{2}\right|\to \frac{1}{\left|1+C_{p}\frac{1}{s}\right|}$$

Obviously, $\left|S_{1}\right|\gg \left|S_{2}\right|\approx 0$ is easy to obtain at low frequency, which means the modified Smith predictor can significantly improve the tracking accuracy of the system and approximately fully compensate for the negative impact of delay. However, although the closed-loop model improves system robustness, the mismatch between the real delay and the model still constrains the value of the controller. The following chapter will continue to analyze how to design feedback and feedforward controllers under stability conditions.

## 4. Stability Analysis and Controller Design

In this section, the stability conditions of the system will be analyzed, and parameter constraints for feedforward and feedback controllers will be given. Based on this, the optimal controller parameters will be obtained. Firstly, the tracking error transfer function of the modified Smith predictor can be rewritten as follows.

(7) $$S_{2}=\frac{E}{R}=\frac{\left(1-C_{f}e^{-\tau_{m}s}\right)+C_{p}\frac{1}{s}\left(1-e^{-\tau_{m}s}\right)}{\left(1+C_{p}\frac{1}{s}\right)\left[1+\frac{\left(C_{p}\frac{1}{s}+C_{f}\right)\left(Ve^{-\tau s}-e^{-\tau_{m}s}\right)}{1+C_{p}\frac{1}{s}}\right]}$$

Since the left part $1+C_{p}∕s$ is absolutely stable due to its negative eigenvalues, the stability conditions of the modified Smith predictor has to satisfy the following equations according to the small-gain theorem.

(8) $${\left\|\frac{\left(C_{p}\frac{1}{S}+C_{f}\right)\left(Ve^{-\tau s}-e^{-\tau_{ms}s}\right)}{1+C_{p}\frac{1}{S}}\right\|}_{\infty }<1$$

Both the classic and modified Smith predictor methods are constructed based on models whose control stability is affected by the model mismatch. Since the high-sampling velocity loop has transformed the platform to have nearly ideal transfer characteristics with V≈1, the main model mismatch is the the uncertainty of delay because of the varying time of the image processing. The feedback controller $C_{p}$ could be set to a proportional controller due to the ideal closed-loop object. To suppress the impact of high-frequency model mismatch, the feedforward controller $C_{f}$ is usually designed to the form of a one-order low-pass filter. Substituting V=1, $C_{p}=k$, $C_{f}=1\left(1+Ts\right)$ and s=jω into Equation (8), for any ω, we can obtain

(9) $$\left|\frac{k+\frac{j\omega }{1+Tj\omega }}{j\omega +k}\left(e^{-j\tau \omega }-e^{-j\tau_{m}\omega }\right)\right|<1$$

Assuming that the bounded uncertainty of delay is $\Delta \tau =\tau_{m}-\tau$, after calculation, Equation (9) is transformed into

(10) $$1-\frac{\left(1+T^{2}\omega^{2}\right)\left(\omega^{2}+k^{2}\right)}{k^{2}+{\left(kT+1\right)}^{2}\omega^{2}}\cdot \frac{1}{2}\leq \cos\left(\omega \Delta \tau \right)$$

Since $\frac{1}{{\left(kT+1\right)}^{2}}\leq \frac{\left(\omega^{2}+k^{2}\right)}{k^{2}+{\left(kT+1\right)}^{2}\omega^{2}}\leq 1$, the stability condition changes to be

(11) $$f_{1}\left(\omega \right)=1-\frac{\left(1+T^{2}\omega^{2}\right)}{{\left(kT+1\right)}^{2}}\cdot \frac{1}{2}\leq \cos\left(\omega \Delta \tau \right)=f_{2}\left(\omega \right)$$

Then, a sufficient condition for Equation (11) is listed as follows.

(12) $$\left|\frac{df_{2}\left(\omega \right)}{d\omega }\right|\leq \left|\frac{df_{1}\left(\omega \right)}{d\omega }\right|$$

By calculating Equation (12), we obtain $\frac{T^{2}\omega }{{\left(kT+1\right)}^{2}}\geq \Delta \tau \sin\left(\omega \Delta \tau \right)$. This condition can hold if

(13) $$\left|\frac{df_{2}^{2}\left(\omega \right)}{d\omega^{2}}\right|\leq \left|\frac{df_{1}^{2}\left(\omega \right)}{d\omega^{2}}\right|$$

Then, the following condition can be derived

(14) $$k+\frac{1}{T}\leq \frac{1}{\Delta \tau \sqrt{\left|\cos\left(\omega \Delta \tau \right)\right|}}$$

Finally, it can be concluded, to obtain asymptotic stability, the following condition should be satisfied

(15) $$k+\frac{1}{T}\leq \frac{1}{\Delta \tau }$$

In Equation (15), if 1/T=0, it means that the feedforward branch does not exist in the closed loop and the stability condition becomes k≤1∕Δτ. In contrast, if k=0, it means that only the feedforward branch works in the system with the stability condition Δτ≤T.

Regarding how to set the values of $C_{p}$ and $C_{f}$ to obtain the best tracking accuracy, we need to pay attention to the tracking error transfer function of the modified Smith predictor again. Considering that the target of the OTS is usually far away, the angular motion information of the target is mainly concentrated in low frequencies. Therefore, the next focus is mainly on improving the low-frequency performance of the system.

Since the delay time is relatively small, $e^{-\tau s}\approx e^{-\tau_{m}s}\approx 1$ can be considered in the low-frequency domain. Then, substituting $C_{p}=k$, $C_{f}=1/\left(1+Ts\right)$, V=1 and $e^{-\tau s}=e^{-\tau_{m}s}=1$ into Equation (4), the simplified form of $S_{2}$ is listed as below.

(16) $$S_{2}\approx \frac{1-C_{f}}{1+C_{p}\frac{1}{s}}=\frac{Ts^{2}}{\left(1+Ts\right)\left(s+k\right)}$$

By substituting s=jω into the equation, we obtain its amplitude

(17) $$\left|S_{2}\right|\approx \frac{\omega^{2}}{\sqrt{\frac{1}{T^{2}}+\omega^{2}}\sqrt{k^{2}+\omega^{2}}}$$

Since in low frequency, we can treat ω≪1∕T and ω≪k, then

(18) $$\begin{array}{rr} \left|S_{2}\right| & \;\approx \frac{\omega^{2}}{\sqrt{\frac{1}{T^{2}}+\omega^{2}}\sqrt{k^{2}+\omega^{2}}}\approx \frac{\omega^{2}}{\frac{1}{T}\cdot k}\geq \frac{\omega^{2}}{{\left(\frac{1}{T}+k\right)}^{2}}\geq \omega^{2}\Delta \tau^{2} \end{array}$$

When 1∕T=k=0.5/Δτ, the equal sign holds and $\left|S_{2}\right|$ obtains the minimum value at low frequency. That is to say, when 1∕T=k=0.5/Δτ, the system has the optimal tracking performance.

## 5. Experimental Verification

The experimental apparatus implementing the OTS is shown in Figure 5, in which there are two fast steering mirror systems. The motion of both mirrors are coupled to the optical path. The fast steering mirror carrying the laser source is a tracking mirror used as the controlled object; the fast steering mirror that reflects laser to the receiver is a target mirror used to simulate target motion. Since the experimental apparatus is a two-axis symmetric system, we take one axis into consideration. A high-bandwidth fiber-optics gyro is used to build the velocity closed loop. In order to facilitate experimental verification, the phase-sensitive demodulator (PSD) replaces the CCD to receive the light and provide the position error to control the tracking mirror. The PSD could run at the sampling rate of 50 Hz with an artificially added delay of about one frame. The gyro could run at the rate of 5000 Hz.

The velocity open-loop transfer function of the fast steering mirror is composed of a differential link, a mechanical resonance link and an inertial link, and is shown below.

(19) $$G_{v}=\frac{K_{v}s}{\frac{s^{2}}{\omega_{n}^{2}}+\frac{2\xi }{\omega_{n}}s+1}\cdot \frac{1}{T_{e}s+1}$$

**Figure 6:**
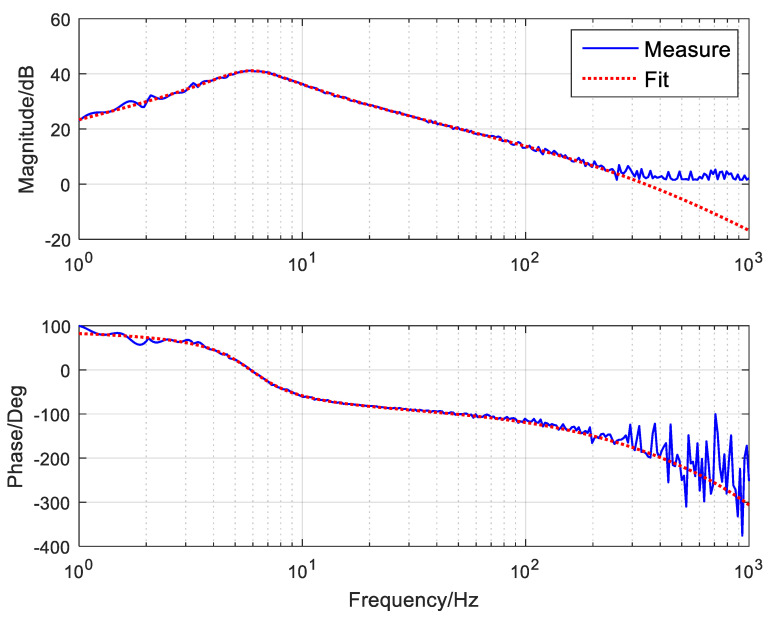
The velocity open-loop bode curve of the platform.

In Equation (19), $K_{v}$ is the gain of the object, $\omega_{n}$ is the natural frequency, ξ is the damping ratio and $T_{e}$ is the electrical constant. To obtain the model parameters of the system, we first need to obtain the velocity open-loop bode curve of the system through the frequency sweep method, as shown in Figure 6. The blue curve represents the actual measurement, and the red represents the fitting curve. By frequency fitting, we can determine the approximate parameters of the characteristics of open-loop objects, as shown in Equation (20).

(20) $${\tilde{G}}_{v}=\frac{2.3s}{0.00072s^{2}+0.0202s+1}\cdot \frac{1}{0.0005s+1}$$

**Figure 7:**
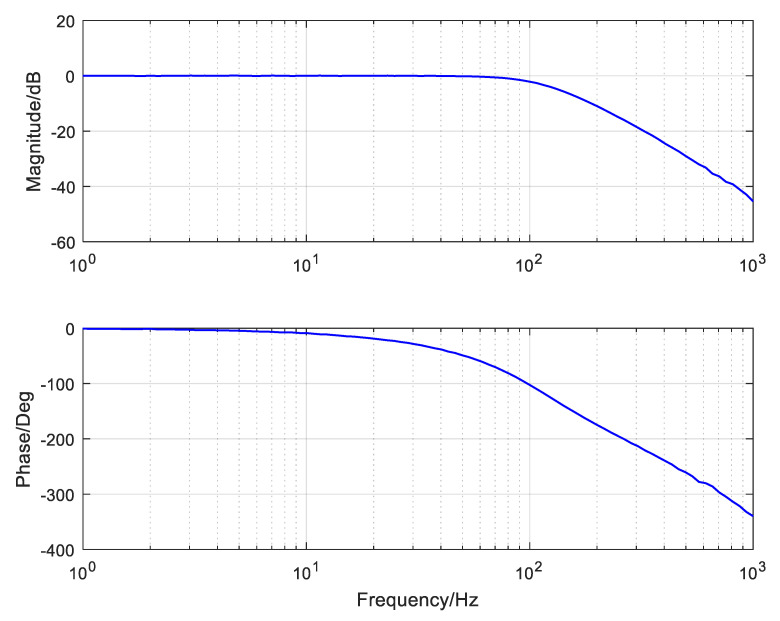
The velocity closed-loop bode curve of the platform.

Based on ${\tilde{G}}_{v}$, we can use the zero-pole elimination method to design a velocity controller. The velocity closed-loop bode curve of the system is exhibited in Figure 7. It can be seen that the velocity closed loop could eliminate the open-loop object resonance and make the inner-loop bandwidth exceed 100 Hz, which is much higher than the bandwidth of the outer loop. Therefore, the velocity closed loop can be regarded as an ideal object in a wide frequency domain, and the open-loop position object changes to be an integral link plus a delay item represented by ${\tilde{G}}_{p}=e^{-0.02s}/s$.

Usually, the uncertainty of delay can be obtained by running the system in different complexity backgrounds. In this experiment, for the convenience of verifying the algorithm, the delay uncertainty was artificially set with Δτ/τ=50%. According to the derived stability condition, the feedback controller of the classic Smith predictor method can be designed as $C_{p}=1/\Delta \tau =1/\left(\tau \cdot 50\%\right)=100$. For the modified Smith predictor method, based on the optimal performance condition, the parameters of the feedback and feedforward controllers should be set to 1∕T=k=0.5/Δτ=50.

**Figure 8:**
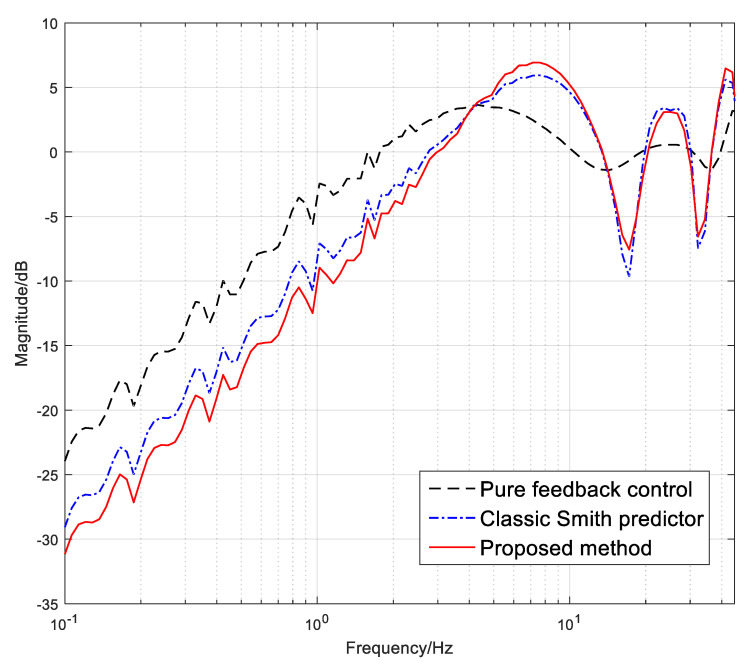
The error rejection ratio with different control methods. It is obtained by comparing the error value to the input signal from 1 Hz to 50 Hz.

**Figure 9:**
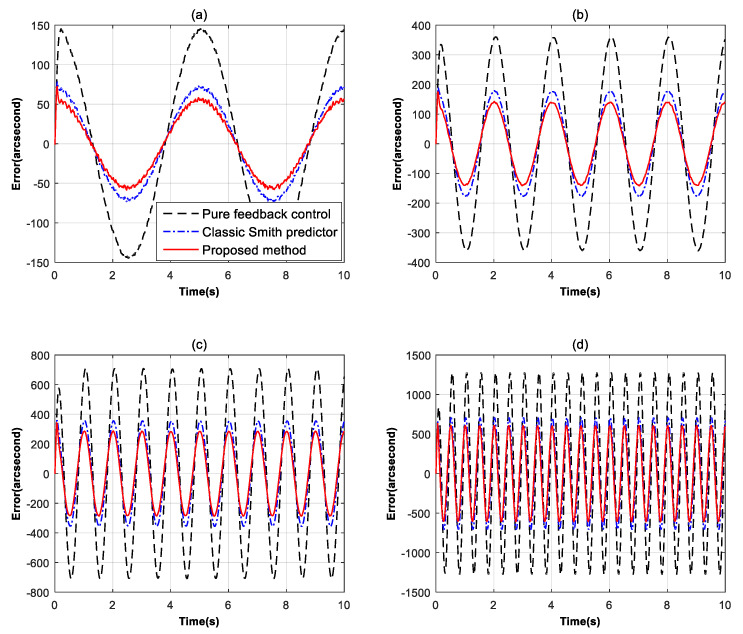
The residual error curves in different frequencies. (**a**) error at 0.2 Hz; (**b**) error at 0.5 Hz; (**c**) error at 1 Hz; (**d**) error at 2 Hz.

To evaluate the tracking performance under different control methods, we used the error suppression ratio as the main evaluation indicator, which is obtained by comparing the error value to the input signal. A smaller error suppression ratio represents stronger tracking performance. Figure 8 exhibits the error suppression ratio curves of the pure feedback control, the classic Smith predictor and the modified Smith predictor by the pseudo feedforward control. It can be seen that the Smith predictor’s ability to separate time delays has brought a −4 dB improvement to the error suppression capability. However, although the classic Smith predictor could promote the tracking ability compared with the feedback control, the improvement is still not enough due to the limitations in the model mismatch. After the proposed modified Smith predictor is added into the system, compared with the classic Smith predictor, the tracking ability of the system has continued to improve by approximately −2 dB below 4 Hz. Although the proposed method does not improve the high-frequency performance, it still meets our requirements as our main focus is on the low-frequency performance of the system. Figure 9 shows the comparison of the real time-domain tracking errors with given target signals in different frequencies. Obviously, with the proposed way, the residual error is significantly reduced in the low-frequency domain.

Figure 9 and Table 1 show the comparison of the real time-domain tracking errors with given target signals in different frequencies. The system receives sine motion signals ranging from 0.2 Hz to 2 Hz with a 1000 arcsec amplitude and tracking errors under different control methods are recorded. Obviously, with the proposed method, the residual error is further reduced in the low-frequency domain which is consistent with the previous theoretical analysis. At this point, the proposed method is close to reaching the tracking performance limit of the system without additional hardware costs.

## 6. Conclusions

In this paper, we proposed a modified Smith predictor by a pseudo feedforward control for the optoelectronic tracking system, which has stronger tracking ability compared to the classic Smith predictor control. Due to the existence of model mismatch, in order to ensure stability, the classic Smith predictor cannot improve performance by infinitely increasing the controller gain. The proposed modified Smith predictor is first built on the closed-loop model to decrease the influence on the model mismatch of the parameter perturbation. Then, an extra pseudo feedforward branch is added inside the structure of the Smith predictor to improve the error suppression ratio under the limited control bandwidth. Stability conditions and optimal design criteria for feedforward and feedback controllers have been analyzed and provided under delay mismatch circumstances. The proposed method demonstrates substantial improvements in tracking performance across the main frequency band (0.2 Hz to 2 Hz). Specifically, at 1 Hz, the residual error was reduced from 365 arcseconds (using the classic Smith predictor) to 283 arcseconds, a 22.5% improvement. The results indicate that the proposed method fully utilizes its control capabilities, reducing dependency on the model without additional cost, and shows great potential for application in various industrial control systems where delay is a critical factor.

## Figures and Tables

**Table 1:** Maximum residual errors (unit/arcsecond) of different methods at different frequencies.

	0.2 Hz	0.5 Hz	1 Hz	2 Hz
Pure feedback control	141	368	724	1281
Classic Smith predictor	72	182	365	672
Proposed method	56	141	283	620

## Data Availability

Data is unavailable due to privacy.
